# Effect of Atmospheres on Transformation of Heavy Metals during Thermal Treatment of MSWI Fly Ash: By Thermodynamic Equilibrium Calculation

**DOI:** 10.3390/molecules27010131

**Published:** 2021-12-27

**Authors:** Facun Jiao, Xulong Ma, Tao Liu, Chengli Wu, Hanxu Li, Zhongbing Dong

**Affiliations:** 1 School of Chemical Engineering, Anhui University of Science and Technology, Huainan 232001, China; maxulong2022@163.com (X.M.); 2019054@aust.edu.cn (T.L.); chlwu@aust.edu.cn (C.W.); hxli@aust.edu.cn (H.L.); dongzb68@163.com (Z.D.); 2 Institute of Energy, Hefei Comprehensive National Science Center, Hefei 230031, China

**Keywords:** MSWI fly ash, thermal treatment, atmosphere, heavy metal, ash melting

## Abstract

The vaporization behaviors of eight heavy metals (Pb, Zn, Cu, Cd, Cr, Co, Mn, and Ni) in municipal solid wastes incineration (MSWI) fly ash during thermal treatment under air atmosphere (21% O_2_/79% N_2_), an inert atmosphere (100% N_2_), and a reducing atmosphere (50% CO/50% N_2_) were evaluated based on a thermodynamic equilibrium calculation by FactSage 8.1. The results show that the reducing atmosphere promotes the melting of MSWI fly ash, resulting in a more liquid phase than in air or an inert atmosphere. Except for Cd, the formation of liquids can dissolve heavy metals and reduce their vaporization ratio. In the air and inert atmospheres, Pb, Zn, Cu, Co, Mn, and Ni vaporize mainly in the form of metallic chlorides, while Cd volatilizes in the form of metallic Cd (g) and CdO (g). In the reducing atmosphere, Co, Mn, and Ni still vaporize as chlorides. Zn and Cd mainly vaporize in the form of Zn (g) and Cd (g), respectively. In terms of Pb, in addition to its chlorides, the volatiles of Pb contain some Pb (g) and PbS (g). Cr has a low vaporization ratio, accounting for 2.4% of the air atmosphere. Cr, on the other hand, readily reacts with Ca to form water-soluble CrCaO_4_, potentially increasing Cr leaching. Except for Cd, the results of this study suggest that the reducing atmosphere is used for the thermal treatment of MSWI fly ash because it promotes the melting of fly ash and thus prevents heavy metal vaporization.

## 1. Introduction

Fly ash from municipal solid wastes incineration (MSWI) is a hazardous waste because it contains a high concentration of heavy metals, dioxins, and other toxic compounds [1]. Before landfilling, MSWI fly ash must be stabilized. Cement solidification, medicament stabilization, thermal treatment, and other methods of stabilization are current available. Among these, thermal treatment is the most promising method for MSWI fly ash treatment because of the benefits of ash volume reduction, dioxin decomposition, stable slag properties, and low heavy metal leaching [2,3,4,5,6]. Fly ash melts and solidifies into glassy slag during the thermal treatment. Thus, the heavy metals are solidified in the Si-O tetrahedron structure of silicate, highly reducing the leaching ratio of heavy metals [7]. Partial heavy metals in MSWI fly ash, on the other hand, vaporize during thermal treatment, potentially causing secondary pollution. As a result, clarifying the vaporization propensity of heavy metals in MSWI fly ash during thermal treatment is critical for developing technologies to prevent heavy metal vaporization.

The factors affecting the vaporization of heavy metals include the content of chlorine, reaction temperature, the chemical composition of fly ash, etc. Heavy metal vaporization is aided by a high chlorine concentration [8]. This is because metallic chlorides have a higher boiling point than oxides, sulfides, and elements [9]. Furthermore, the mode of occurrence of chlorine is closely related to the heavy metal vaporization. Researchers have extensively investigated the effects of different chlorine-bearing compounds (e.g., NaCl, CaCl_2_, MgCl_2_, and PVC) on the vaporization of heavy metals [10,11,12,13,14,15]. The addition of PVC resulted in the indirect chlorination of Pb and Cd, which means that Cl-containing gas was initially released during the decomposition of the PVC and then reacted with Pb and Cd to form gaseous metallic chlorides. In the case of NaCl, it directly chlorinates Pb and Cd, implying that NaCl reacts with Pb and Cd directly via solid-solid reaction [10]. Yu et al. [15] studied the addition of MgCl_2_ on the vaporization of heavy metals from MSWI fly ash. The MgCl_2_ decomposed and released HCl, which further chlorinates heavy metals forming metallic chlorides. Kageyama et al. [16] investigated the effect of NaCl, KCl, and CaCl_2_ on the vaporization of Pb from CaO-SiO_2_-Al_2_O_3_ molten slag. The vaporization ratio of Pb against the addition of inorganic chlorides follows a descending sequence of CaCl_2_ > NaCl > KCl. The influence of the chemical composition of fly ash on the vaporization of heavy metals is mainly achieved by changing the melting characteristics of fly ash [17,18,19]. SiO_2_ reacts with Al_2_O_3_ and CaO to form eutectic with a low-melting point, improving the ability of glass melts to capture heavy metals and limiting their volatilization [20,21]. Jiao et al. [22] investigated the vaporization of cesium during thermal treatment of the simulated fly ash by adding CaCl_2_. When the treated temperature reaches 1400 °C, a large number of liquids form in the ash, resulting in a lower Cs vaporization ratio. The formation of liquid in treated ash blocks the pore within the ash, preventing cesium vapor from diffusing outward.

In addition, the atmosphere during thermal treatment is another critical factor affecting the vaporization of heavy metals. The atmosphere can alter the ash fusibility and heavy metal species in fly ash, resulting in a variation in heavy metal vaporization behavior. Previous research investigated the effect of oxygen content on cesium vaporization during thermal treatment of MSWI fly ash [22,23]. The results indicate that increase in oxygen content inhibits the vaporization of cesium. Accordingly, the reaction atmospheres are probably influential in the vaporization of heavy metals during thermal treatment of MSWI fly ash. Moreover, the effect of various atmospheres on the vaporization of heavy metals has not been thoroughly investigated. Notably, the migration and transformation of heavy metals during thermal treatment are far from understood. Conventional analysis techniques are unavailable for determination of the mode of the occurrence of heavy metals in the solid, liquid, and gaseous phases during thermal treatment due to their extremely low concentration in each portion, limiting the discovery of the vaporization mechanism of heavy metals. The thermodynamic equilibrium calculation is considered a powerful approach to describe the migration and transformation of heavy metals under different treatment conditions, such as reaction temperatures and atmospheres [24,25]. The vaporization behavior of heavy metals in MSWI fly ash during thermal treatment in various atmospheric conditions (air, inert, and reducing atmospheres) was evaluated using thermodynamic equilibrium calculations in this study. The calculated results of heavy metal vaporization in various atmospheres were validated by experimental observation. The findings can be used to optimize the treatment process, operating parameters, and tail gas clean-up to reduce heavy metal vaporization during thermal treatment.

## 2. Materials and Methods

### 2.1. Properties of MSWI Fly Ash

**Figure 1:**
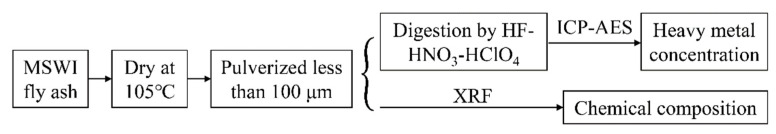
Schematic of the preparation of the MSWI fly ash.

The fly ash was collected from a bag filter in an MSWI plant in China. The preparation procedure of the MSWI fly ash is depicted in Figure 1. The collected ash was subjected to drying in an oven at 105 °C and then pulverized to less than 100 μm. An X-ray fluorescence spectrometer (XRF) was used to determine the chemical composition of fly ash. Inductively coupled plasma-atomic emission spectrometry (ICP-AES) was used to determine the heavy metals in fly ash after digestion with HF-HNO_3_-HClO_4_. The detailed procedure of the acid digestion was introduced elsewhere [9]. The chemical composition as well as the heavy metal concentration are shown in Table 1 and Table 2, respectively. Because of the cleaning of the flue gas with Ca-containing compounds, the fly ash contained a high percentage of CaO (53.7%). Because plastic and kitchen waste are major components of MSW [26], the content of Cl was 22%. The concentrations of Pb and Zn are 1623 mg/kg and 6394 mg/kg, respectively. The concentrations of Cu, Cr, and Mn are between 100 mg/kg and 500 mg/kg, while the concentrations of Cd, Co, and Ni are below 100 mg/kg.

### 2.2. Thermodynamic Equilibrium Calculation

The vaporization of heavy metals and their species in the solid phase, liquid slag, and gaseous phase during thermal treatment of MSWI fly ash were calculated using FactSage 8.1 based on the minimum of Gibbs free energy [27]. The amount of the oxides/elements listed in Table 1 and Table 2 used as inputs was calculated using 100 g MSWI fly ash. The reaction atmospheres included air (21% O_2_/79% N_2_), inert (100% N_2_), and reducing atmospheres (50% CO/50% N_2_). The total gas amount is 6 L used in the calculation. The outputs include solid phase, gas phase and liquid slag. The reaction pressure was 1 atm. The calculation temperature ranged from 600 °C to 1600 °C, with intervals of 100 °C. The database used included FactPs, FToxid, and FTsalt. The fraction of the liquid phase was calculated using a module of FToxid-SLAGA.

### 2.3. Experimental Apparatus and Procedure

**Figure 2:**
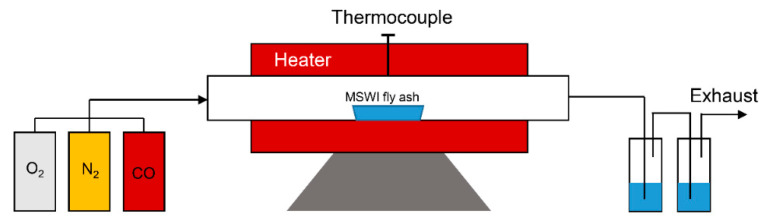
Schematics of the horizontal furnace.

To validate the thermodynamic equilibrium calculation results, heavy metal vaporization experiments were performed in a lab-scale electrically heated horizontal furnace. The schematic of the furnace is shown in Figure 2. Approximately ~2 g MSWI fly ash was loaded in the combustion boat. When the temperature in the furnace reached 1200 °C, the ash-laden boat was inserted into the constant temperature zone. To continuously monitor the reaction temperature, a thermocouple was installed in the middle of the reactor. Two gas scrubbers containing 0.5 mol/L HNO_3_ were employed to clean the reactant gas before exhausting. After a 20 min reaction, the ash was moved out of the reactor and cooled in ambient. The reaction atmospheres were air (21%O_2_/79%N_2_), inert (100%N_2_), and reducing atmospheres (50%CO/50%N_2_). The gas flow rate was 0.3 L/min. The treated MSWI fly ash was pulverized and then subjected to acid digestion using HF-HNO_3_-HClO_4_. The concentration of heavy metals in the final solution was determined by ICP-AES. The vaporization ratio of each metal was calculated according to Equation (1):

(1) $$\mathrm{Vaporization}\;\mathrm{ratio},\;\%=\frac{m_{1}-m_{2}}{m_{1}}\times 100$$

where m_1_ and m_2_ denote the amount of each heavy metal before and after the thermal treatment, respectively.

## 3. Results and Discussion

### 3.1. Effect of Atmosphere on the Fusibility of MSW Fly Ash

**Figure 3:**
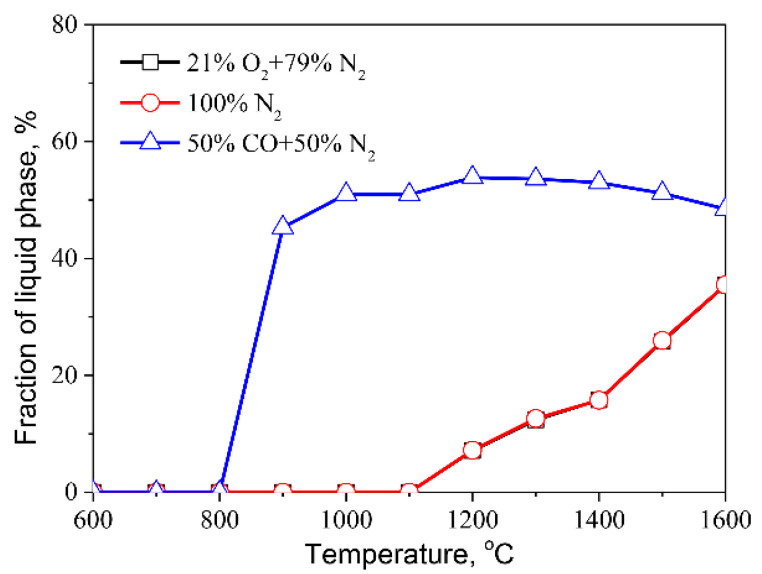
Liquid fraction in the MSWI ash as functions of atmospheres and temperatures.

The fraction of liquid phase in MSWI fly ash treated at various temperatures and atmospheres is first calculated because the fly ash fusibility during thermal treatment plays an important role in the migration and transformation of heavy metals [22,28,29]. The outcome of the calculation are depicted in Figure 3. The change curves of the liquid fraction in fly ash as a function of temperature almost overlap in the air and inert atmospheres, indicating that the air and inert atmospheres do not affect the fly ash fusibility. The liquids in the fly ash begin to form at 1100 °C, and the fraction of the liquid phase is around 35.5% at 1600 °C. In a reducing atmosphere, the fly ash begins to melt at 800 °C, and the fraction of liquid phase reaches 51% at 1000 °C. When compared to air or inert atmospheres, the results indicated that reducing atmosphere facilitates the melting of fly ash.

### 3.2. Transformation of Heavy Metals in Different Atmospheres

#### 3.2.1. Lead (Pb)

**Figure 4:**
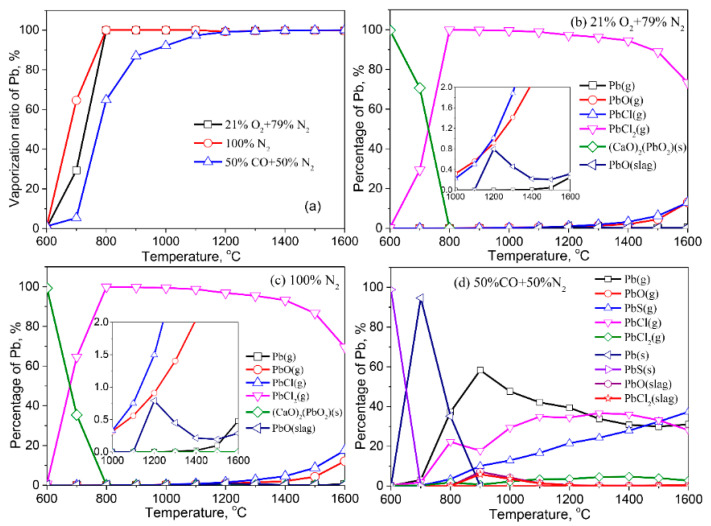
Vaporization behavior of Pb under different atmospheres. (**a**) Vaporization ratio of Pb as a function of temperature in different atmospheres; (**b**) Distribution of Pb in 21%O_2_ plus 79%N_2_; (**c**) Distribution of Pb in 100%N_2_; (**d**) Distribution of Pb in 50%CO plus 50%N_2_.

The vaporization behaviors of Pb in various atmospheres are shown in Figure 4. The vaporization ratio of Pb in the three atmospheres reaches 100% when the temperature exceeds 1200 °C (Figure 4a). Nonetheless, when the temperature is below 1200 °C, the vaporization ratio of Pb is significantly affected by the atmosphere. Compared with the air and inert atmospheres, the reducing atmosphere delays Pb vaporization.

In the air atmosphere, Pb preferentially combines with Ca to form stable (CaO)_2_(PbO_2_) (s) at <600 °C (Figure 4b). The formed (CaO)_2_(PbO_2_)(s) gradually changes to gaseous PbCl_2_ (g) at 600–800 °C. This result indicates Cl in fly ash induced the vaporization of Pb, which is in line with the experimental observations from other works in the literature reported [10,29,30]. With increasing temperature, a small amount of Pb vaporizes as PbO (g) and PbCl (g). When the temperature reaches 1100 °C, tiny amounts of Pb are dissolved in the liquid slag in the form of PbO (slag) due to the formation of the liquid phase in the fly ash. The Pb-bearing species in the inert atmosphere are consistent with those in the air atmosphere, but at the temperature of 700 °C, more (CaO)_2_(PbO_2_) (s) transforms to PbCl_2_ (g) under an inert atmosphere (Figure 4c), resulting in the vaporization ratio of Pb in the inert atmosphere being higher than that in the air atmosphere.

The Pb-bearing species among the solid phase, liquid slag, and gaseous phase in the reducing atmosphere differ significantly from those in the air and inert atmospheres. When the temperature is below 600 °C, Pb mainly exists in the fly ash as PbS (s), which further transforms into Pb (s) at 700 °C. When the temperature is above 700 °C, Pb (s) converts to gaseous Pb (g), PbCl (g), and PbS (g). A small amount of Pb is dissolved in the liquid slag in the form of PbO (slag) and PbCl_2_ (slag), and its content in the liquid slag decreases with an increasing temperature (Figure 4d).

#### 3.2.2. Zinc (Zn)

**Figure 5:**
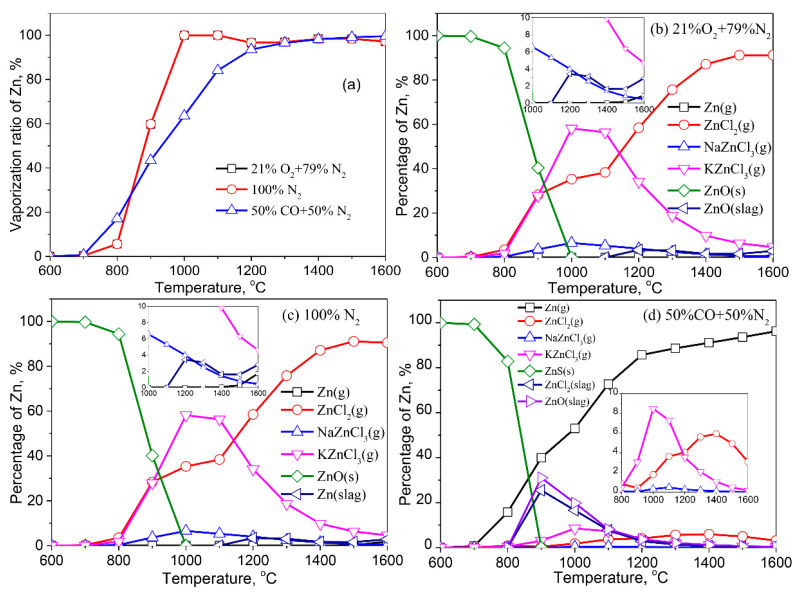
Vaporization behavior of Zn under different atmospheres. (**a**) Vaporization ratio of Zn as a function of temperature in different atmospheres; (**b**) Distribution of Zn in 21%O_2_ plus 79%N_2_; (**c**) Distribution of Zn in 100%N_2_; (**d**) Distribution of Zn in 50%CO plus 50%N_2_.

Figure 5 demonstrates the vaporization of Zn in various atmospheric conditions. As shown in Figure 5a, the vaporization ratio of Zn as a function of temperature in the air and inert atmospheres was nearly identical, indicating that the two atmospheres have a similar effect for Zn vaporization. The vaporization behavior of Zn in a reducing atmosphere is quite different from that in an inert atmosphere or air. When the temperature is less than 850 °C, Zn vaporizes more easily in a reducing atmosphere than in air or inert atmospheres. When the temperature rises above 850 °C, the result is the opposite.

Zn in the fly ash exists in the form of ZnO (s) at <800 °C in the air atmosphere (Figure 5b). When the temperature is higher than 800 °C, ZnO (s) begins to transform into gaseous ZnCl_2_ (g), KZnCl_3_ (g), and NaZnCl_3_ (g). A small amount of ZnO is dissolved in the liquid slag at >1100 °C, and its content in the slag is subject to an initial increase and then a decrease with the rise of temperature. The vaporization behavior of Zn in an inert atmosphere is nearly consistent with that in an air atmosphere. In the reducing atmosphere, however, the migration and transformation behavior of Zn changes significantly (Figure 5d). ZnS (s) is a stable species in the fly ash at <700 °C. With the increase of temperature, partial ZnS (s) transforms into gaseous Zn (g), and the content of Zn (g) gradually increases with the increase of temperature. At 800 °C, Zn is partially dissolved in the liquid slag in the form of ZnCl_2_ (slag) and ZnO (slag). The proportion of Zn in the liquid slag gradually decreases because it vaporizes as Zn (g), KZnCl_3_ (g), ZnCl_2_ (g), and NaZnCl_3_ (g) at >900 °C.

#### 3.2.3. Cadmium (Cd)

**Figure 6:**
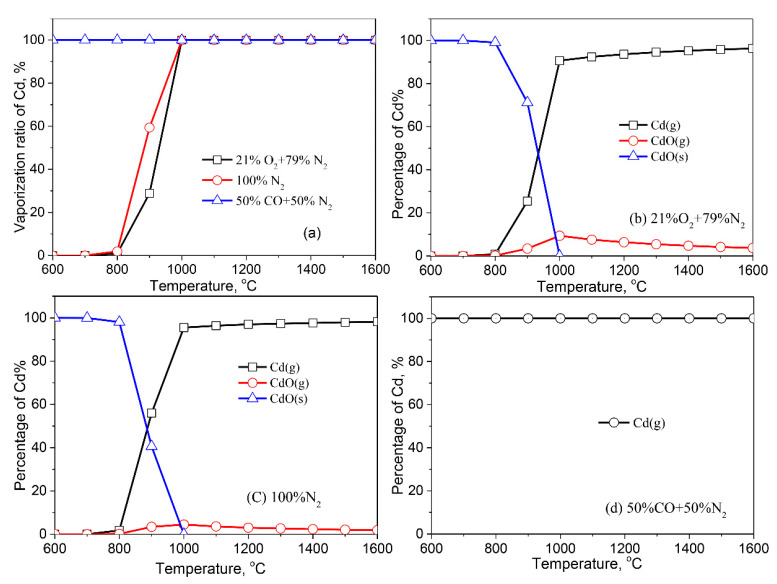
Vaporization behavior of Cd under different atmospheres. (**a**) Vaporization ratio of Cd as a function of temperature in different atmospheres; (**b**) Distribution of Cd in 21%O_2_ plus 79%N_2_; (**c**) Distribution of Cd in 100%N_2_; (**d**) Distribution of Cd in 50%CO plus 50%N_2_.

The vaporization behaviors of Cd are shown in Figure 6. Cd vaporizes completely at 600–1600 °C in the reducing atmosphere, which results from Cd in the fly ash being reduced to metallic Cd with a melting point of 321 °C (Figure 6d). In the air and inert atmospheres, Cd vaporizes from 700 °C; its vaporization ratio increases rapidly as the temperature is higher than 800 °C. The vaporization ratio of Zn is up to 100% at 1000 °C. The Cd-species present in the air atmosphere are identical to those found in the inert atmosphere. CdO (s) is a dominant Cd-bearing compound in the fly ash at <800 °C. An increase in the temperature results in the transformation of Cd into Cd (g) and CdO (g). The inert atmosphere favors the conversion of CdO (s) into Cd (g) (Figure 6c) at 800–1000 °C, resulting in a higher vaporization ratio of Cd in the inert atmosphere than that in the air atmosphere.

#### 3.2.4. Copper (Cu)

**Figure 7:**
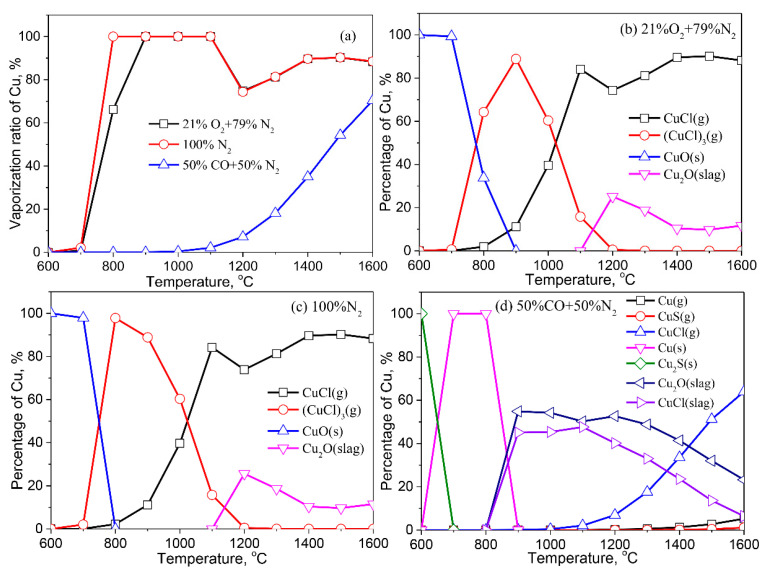
Vaporization behavior of Cu under different atmospheres. (**a**) Vaporization ratio of Cu as a function of temperature in different atmospheres; (**b**) Distribution of Cu in 21%O_2_ plus 79%N_2_; (**c**) Distribution of Cu in 100%N_2_; (**d**) Distribution of Cu in 50%CO plus 50%N_2_.

Figure 7 summarizes the calculation results for the vaporization behaviors of Cu in various atmospheres during thermal treatment. Cu vaporizes from 700 °C both in air and inert atmospheres. The vaporization ratio of Cu reaches 100% at 800 °C in inert atmospheres and at 900 °C in air atmospheres, respectively. The vaporization ratio of Cu increases initially and then decreases at >900 °C. Cu vaporizes from 900 °C in the reducing atmosphere, and its vaporization ratio increases with the increase of temperature. When compared to air or an inert atmosphere, the reducing atmosphere inhibits the vaporization of Cu (Figure 7a).

Cu in fly ash exists in CuO (s) in the air and inert atmospheres at <700 °C. With the increase of temperature, Cu is released to the gas phase as CuCl (g) and (CuCl)_3_ (g). Partial Cu dissolves in the liquid slag as Cu_2_O (slag) at >1100 °C, resulting in a decrease of the vaporization ratio of Cu at 1200 °C. When the temperature is higher than 1200 °C, Cu dissolved in the liquid slag releases in the form of CuCl (g) (Figure 7b,c). In the reducing atmosphere, Cu exists in the form of Cu_2_S (s) at low temperatures. The proportion of metallic Cu (s) reaches 100% at 700–800 °C. Cu dissolves in liquid slag as CuO (slag) and CuCl (slag) at >800 °C, and its content in the liquid slag under a reducing atmosphere is much higher than that in the air or inert atmospheres. These findings are related to more liquids formed in the fly ash in the reducing atmosphere (Figure 3). The content of Cu in the liquid slag increases initially and then decreases with the increase of temperature due to the formation of CuCl (g).

#### 3.2.5. Chromium (Cr)

**Figure 8:**
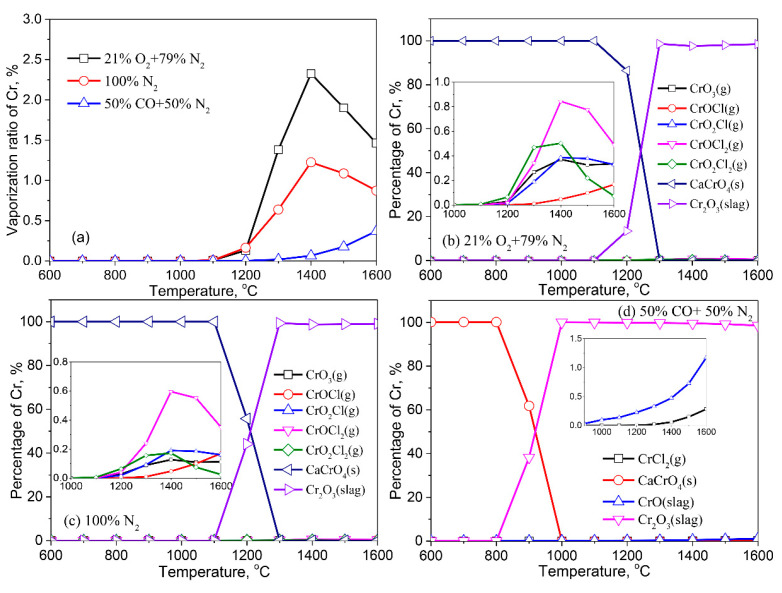
Vaporization behavior of Cr under different atmospheres. (**a**) Vaporization ratio of Cr as a function of temperature in different atmospheres; (**b**) Distribution of Cr in 21%O_2_ plus 79%N_2_; (**c**) Distribution of Cr in 100%N_2_; (**d**) Distribution of Cr in 50%CO plus 50%N_2_.

Figure 8 shows the vaporization of Cr in various atmospheres. The effect of the atmosphere on the vaporization of Cr follows a descending sequence of air atmosphere > inert atmosphere > reducing atmosphere. Cr vaporizes at around 1200 °C. With the increase of temperature, the vaporization of Cr increases initially and then decreases in air and inert atmospheres. In contrast, the vaporization ratio of Cr increases with an increasing temperature in the reducing atmosphere. In comparison to the previously mentioned elements of Pb, Zn, Cu, and Cd, the maximum vaporization ratio is relatively low, accounting for only 2.3% at 1400 °C in the air atmosphere (Figure 8a).

Cr-bearing species in the solid phase under different atmospheres are the same. Cr reacts with Ca in the fly ash to form CaCrO_4_ (s) in air and inert atmospheres at <1200 °C (Figure 8b,c). Hu and Chen et al. investigated the reaction mechanisms between Cr and Ca during thermal treatment of MSWI fly ash and coal combustion [2,31]. The experimental results agree with the thermodynamic equilibrium calculation used in this study. The formation of water-soluble CaCrO_4_ (s) potentially result in a higher Cr leaching ratio in the treated ash, negatively impacting the environment. As the temperature rises to 1100 °C, Cr begins to dissolve in the liquid slag in the form of Cr_2_O_3_ (slag). Meanwhile, a small amount of Cr vaporizes to the gas phase with CrOCl_2_ (g), CrO_2_Cl_2_ (g), CrO_3_ (g), CrO_2_Cl (g) and CrOCl (g). Under the reducing atmosphere, Cr begins to dissolve in the liquid slag in the form of Cr_2_O_3_ (slag) and CrO (slag) at >800 °C (Figure 8d), which is also related to the formation of more liquids in the fly ash in a reducing atmosphere. It can be concluded that in a reducing atmosphere, the solidification of Cr in slag can occur at relatively low temperatures, resulting in a lower leaching ratio. As the temperature rises, a trace amount of Cr is released into the gas phase in the form of CrCl_2_ (g).

#### 3.2.6. Cobalt (Co)

**Figure 9:**
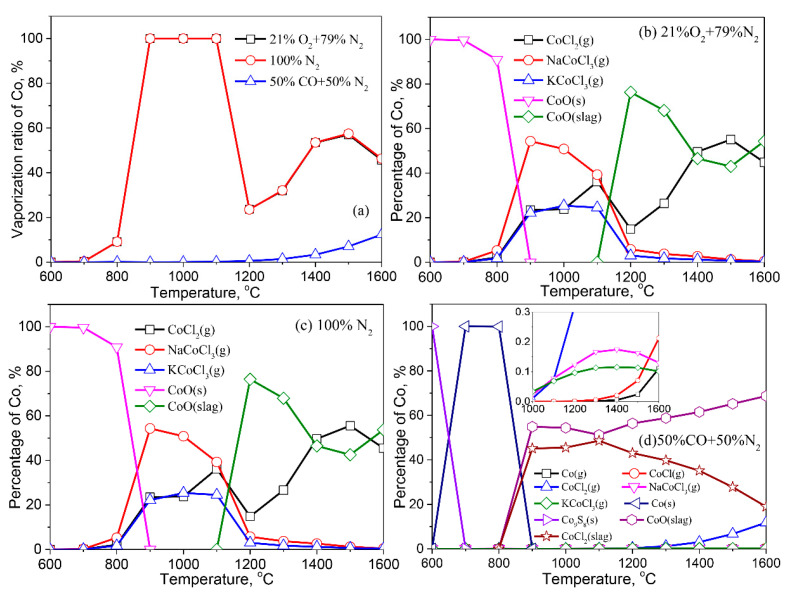
Vaporization behavior of Co under different atmospheres. (**a**) Vaporization ratio of Co as a function of temperature in different atmospheres; (**b**) Distribution of Co in 21%O_2_ plus 79%N_2_; (**c**) Distribution of Co in 100%N_2_; (**d**) Distribution of Co in 50%CO plus 50%N_2_.

The vaporization behaviors of Co are shown in Figure 9. The variation of the Co vaporization ratio as a function of temperature is nearly identical in air and inert atmospheres. The volatilization ratio of Co reaches 100% at 900–1100 °C. Co volatilizes in the reducing atmosphere at 1200 °C, and the vaporization ratio gradually increases with increasing temperature. The reducing atmosphere inhibits Co vaporization, relative to air and inert atmospheres (Figure 9a).

Co exists in the form of CoO (s) under the air and inert atmosphere at <800 °C. With the increase of temperature, Co associates with Na, K, and Cl in the fly ash to form gaseous CoCl_2_ (g), NaCoCl_3_ (g), and KCoCl_3_ (g). As the temperature is higher than 1100 °C, partial Co dissolves in the liquid slag, resulting in the decrease of the vaporization ratio of Co. An increase in temperature causes the vaporization of Co from the liquid phase as CoCl_2_ (g). In the reducing atmosphere, Co in the fly ash at low temperature is mainly in the form of sulfides, while the content of metallic Co is up to 100% at 700–800 °C. Co dissolves in the liquid slag with CoCl_2_ (slag) and CoO (slag) at >800 °C. When the temperature is raised to 1000 °C, a minor fraction of Co is released into the gaseous phase in the form of CoCl_2_ (g), NaCoCl_3_ (g), KCoCl_3_ (g), CoCl (g), and Co (g).

#### 3.2.7. Manganese (Mn)

**Figure 10:**
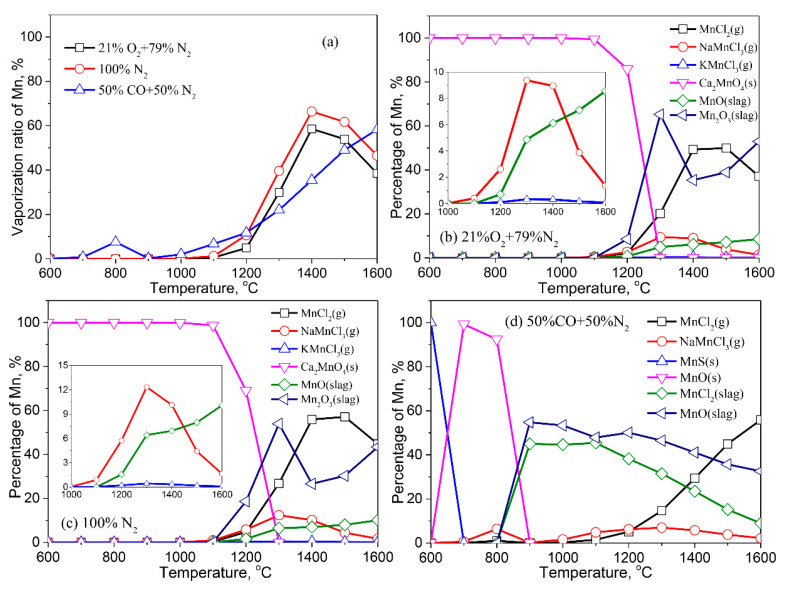
Vaporization behavior of Mn under different atmospheres. (**a**) Vaporization ratio of Mn as a function of temperature in different atmospheres; (**b**) Distribution of Mn in 21%O_2_ plus 79%N_2_; (**c**) Distribution of Mn in 100%N_2_; (**d**) Distribution of Mn in 50%CO plus 50%N_2_.

Figure 10 depicts the vaporization ratio of Mn as well as its distribution in the solid, liquid, and gaseous phases. In air and an inert atmosphere, the vaporization ratio of Mn increases initially and then decreases as temperature rises. In comparison to the air atmosphere, the inert atmosphere is more favorable for the vaporization of Mn. A small amount of Mn volatilizes to the gas phase in the reducing atmosphere between 600–900 °C, and its volatilization increases first, then decreases. When the temperature rises above 900 °C, the vaporization ratio of Mn rises gradually.

Mn reacts with Ca to form Ca_2_MnO_4_ (s) at <1100 °C in air and an inert atmosphere (Figure 10b,c). Partial Mn dissolves into the liquid slag with Mn_2_O_3_ (slag) and MnO (slag) at >1100 °C; another part of Mn vaporizes into gaseous in the form of MnCl_2_ (g), NaMnCl_3_ (g), and KMnCl_3_ (g). In the reducing atmosphere (Figure 10d), the dominant Mn-bearing compound in the fly ash is MnS at low temperature, which transforms to MnO (s) at 700–800 °C. When the temperature is higher than 800 °C, partial Mn dissolves into the liquid slag as MnCl_2_ (slag) and MnO (slag), which vaporizes into gaseous in the form of MnCl_2_ (g) and NaMnCl_3_ (g) with the increase of temperature.

#### 3.2.8. Nickle (Ni)

**Figure 11:**
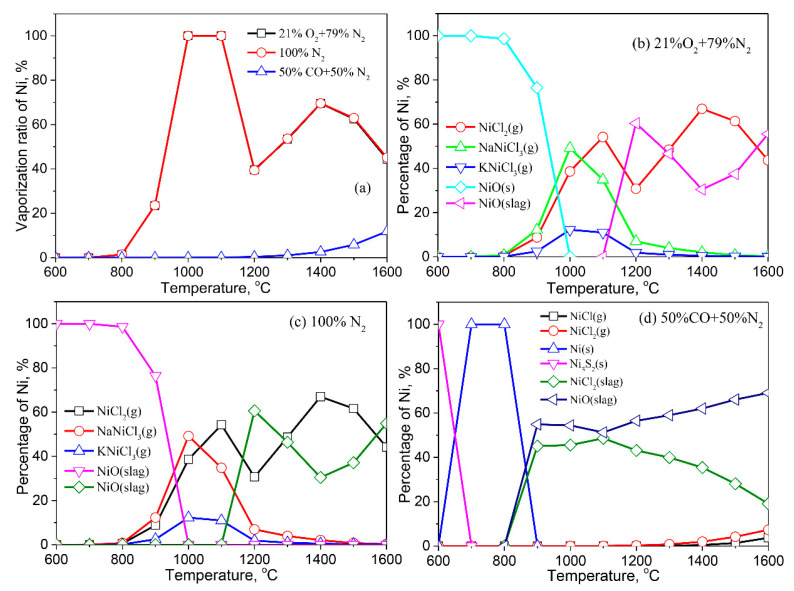
Vaporization behavior of Ni under different atmospheres. (**a**) Vaporization ratio of Ni as a function of temperature in different atmospheres; (**b**) Distribution of Ni in 21%O_2_ plus 79%N_2_; (**c**) Distribution of Ni in 100%N_2_; (**d**) Distribution of Ni in 50%CO plus 50%N_2_.

Figure 11 shows the vaporization of Ni in various atmospheres. The effect of a reaction atmosphere and temperature on Ni is similar to that of Co. Co vaporizes from 800 °C in air and inert atmospheres, and its vaporization ratio reaches 100% at 1000 °C. Ni begins to vaporize at 1200 °C in the reducing atmosphere. The vaporization ratio of Ni rises as the temperature rises. The reducing atmosphere facilitates the vaporization of Ni, compared with that in air and in inert atmospheres.

NiO (s) is a stable Ni-bearing compound in the fly ash at low temperature in the air and inert atmospheres (Figure 11b,c). When the temperature is higher than 800 °C, Ni reacts with Na, K, and Cl to form NaNiCl_3_ (g), NiCl_2_ (g), and KNiCl_3_ (g). Ni dissolves into the liquid slag at 1100 °C, reducing the vaporization ratio of Ni. In the reducing atmosphere, Ni_3_S_2_ (s) is a main Ni-bearing species in the fly ash at low temperature, and it is reduced to metallic Ni (s) at 700–800 °C. When the temperature exceeds 800 °C, Ni dissolves into the liquid slag as NiCl_2_ (slag) and NiO (slag). With the temperature further increasing, the content of NiCl_2_ (slag) in the liquid slag decreases because partial NiCl_2_ (slag) vaporizes in the form of NiCl_2_ (g) and NiCl (g).

### 3.3. Validation of the Vaporization of Heavy Metals

**Figure 12:**
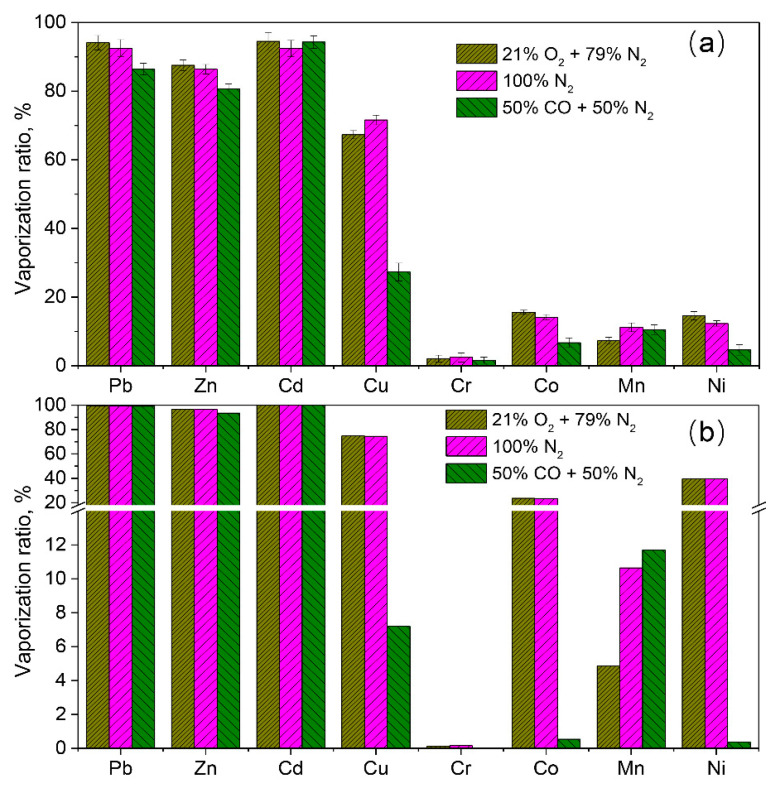
Vaporization ratio of heavy metals during thermal treatment of MSWI fly ash at 1200 °C under different atmospheres. (**a**) Experimental results; (**b**) Results from the thermodynamic equilibrium calculation.

In Section 3.2, the vaporization behavior of heavy metals was predicted using thermodynamic equilibrium calculation in an air atmosphere (21% O_2_/79% N_2_), an inert atmosphere (100% N_2_), and a reducing atmosphere (50% CO/50% N_2_). To validate the reliability of the calculation results, the vaporization of the eight heavy metals was examined in an electrically heated horizontal furnace at 1200 °C under air, inert, and reducing atmospheres, respectively. Their vaporization ratio of each metal against the treated atmosphere is shown in Figure 12a. The vaporization ratio of each metals based on the thermodynamic equilibrium calculation was also shown in Figure 12b for comparison. Regardless of the treated atmosphere, the four heavy metals Cr, Co, Mn, and Ni exhibit a lower vaporization propensity than the heavy metals Pb, Zn, Cd, and Cu. This result is in agreement with that of the thermodynamic equilibrium calculation. The reaction atmosphere is influential in the vaporization of these heavy metals. The reducing atmosphere inhibits the vaporization of Pb, Zn, Cu, Cr Co, and Ni. The vaporization of Cd is independent on the reaction atmospheres because Cd was not dissolved in the liquid slag according to the calculation results in Figure 6. With respect to the vaporization ratio of each metals between the experimental and calculation results, the vaporization ratios in the experimental results are generally lower than that of thermodynamic equilibrium calculation. Take Pb and Cd as an example: the vaporization ratio of Pb and Cd in an air atmosphere is close to 100% based on the thermodynamic equilibrium calculation, whereas their vaporization ratios are around 94% according to the experimental observation. A possible reason for this discrepancy is that apart from thermodynamics, kinetic control is another governing factor for the vaporization of heavy metals in MSWI fly ash during thermal treatment. Even so, the thermodynamic equilibrium calculation results are still significant in guiding the thermal treatment of MSWI fly ash. According to the findings in this study, a reducing atmosphere is proposed during the thermal treatment of MSWI fly ash, since much heavy metals such as Pb, Zn, Cu, Co, and Ni will be solidified in the treated ash.

## 4. Conclusions

The vaporization behaviors of heavy metals in MSWI fly ash during thermal treatment in air, inert, and reducing atmospheres were evaluated using thermodynamic equilibrium calculations. The calculation results were confirmed by experimental observation. The following are the primary conclusions:

(1) Except for Cd, the formation of liquid slag in the treated ash dissolves the heavy metals, lowering their vaporization ratio. With the increase of the reaction temperature, the dissolved heavy metals in the liquid slag are released into the gaseous phase.
(2) The vaporization of Pb, Zn, Cu, Cr, Co, and Ni is inhibited by a reducing atmosphere when compared to air and inert atmospheres. The main reason is attributed to a large amount of liquid slag formed in the reducing atmosphere. As a result, the vaporization ratio of heavy metals is reduced.
(3) The formation of metallic chlorides causes the vaporization of Pb, Zn, Cu, Co, Mn, and Ni during the thermal treatment of MSWI fly ash in the air and inert atmosphere. Cd vaporizes in the form of Cd or CdO. In the reducing atmosphere, Zn and Cd mainly vaporize in the form of Zn (g) and Cd (g). Concerning Pb, apart from its chloride, the formation of Pb (g) and PbS (s) are also contributed to the vaporization of Pb.
(4) Cr has the lowest vaporization propensity among these heavy metals in the fly ash during thermal treatment. However, Cr readily reacts with Ca to form water-soluble CrCaO_4_ (s), potentially causing the leaching risk of Cr in the treated ash.
(5) The calculated results for the vaporization of heavy metals as a function of reaction atmosphere matched the experimental observation. As a result, a reducing atmosphere is proposed as a method for preventing the vaporization of heavy metals during the thermal treatment of MSWI fly ash.

## Figures and Tables

**Table 1:** Chemical composition of MSWI fly ash, %.

Species	SiO_2_	Al_2_O_3_	Fe_2_O_3_	CaO	MgO	K_2_O	Na_2_O	TiO_2_	SO_3_	Cl
Content	3.8	2.54	1.12	53.7	1.09	3.31	5.42	0.72	4.18	22

**Table 2:** Concentration of heavy metals in MSWI fly ash, mg/kg.

Elements	Pb	Zn	Cu	Cd	Cr	Mn	Co	Ni
Concentration	1623	6394	424	57	241	380	35	50

## Data Availability

Not applicable.

## Sample Availability

Samples of the compounds are not available from the authors.
